# Information Transport across a Membrane

**DOI:** 10.1371/journal.pbio.0020168

**Published:** 2004-06-15

From a biochemical perspective, a living cell is a collection of molecules jampacked into a confined space by a flexible barrier, called the plasma membrane. A diverse array of proteins embedded in the plasma membrane act as conduits between the cell interior and its external environment, conveying nutrients, metabolites, and information. The life of a cell—as well as that of any multicellular organism—depends on a cell's ability to communicate with its neighbors, both near and far. One way cells do this is with transmembrane receptors outfitted with both extracellular and intracellular domains that mediate information flow between the cell's external and internal environment. One class of transmembrane receptors, called integrin receptors, specializes in interacting with and binding to other cells and the extracellular matrix, a complex of molecules surrounding cells that provides structural support. By integrating various components of the extracellular matrix, integrins (also known as adhesion receptors), play an important role in such diverse processes as cell differentiation, programmed cell death, wound healing, and metastasis.[Fig pbio-0020168-g001]

**Figure pbio-0020168-g001:**
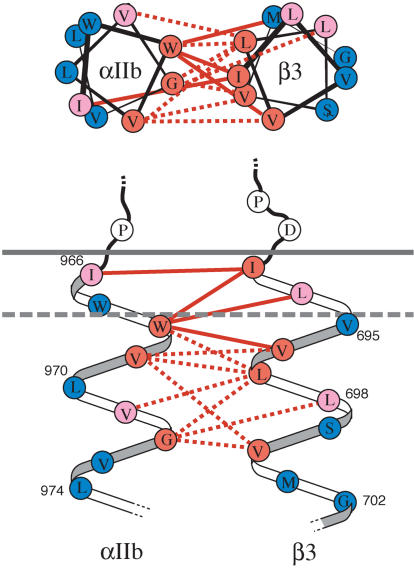
Association between integrin α and β subunit transmembrane domains

Integrins can be regulated by signals within the cell to bind to their ligands with either low or high affinity. While a multitude of integrin ligands have been identified and the general mechanics of both the extracellular and intracellular domains of these receptors are known, exactly how a signal crosses the receptor's transmembrane segment to regulate affinity has remained obscure. Now, Bing-Hao Luo, Timothy Springer, and Junichi Takagi have taken a mutational approach to shed light on the inner workings of the transmembrane segment and to explain how it transmits information.

Much of what we know about the function of integrins has come from studying the crystal structures and models obtained from structural analysis. These analyses have generated information not only about the structure and composition of the extracellular and intracellular domains of integrins, but also about the conformational changes that accompany signaling events. Integrins contain a large extracellular domain, a transmembrane segment, and a relatively short intracellular “tail.” Integrins are heterodimers—molecules that contain two subunits composed of different amino acids—made up of an α chain and a β chain. Tight association of the two subunits is associated with an inactive, or low-affinity, state of the extracellular ligand-binding domain. Separation of the intracellular subunits is associated with a dramatic conformational change and activation of the extracellular domain, changing a bent structure with a downward-pointing ligand-binding site into an extended one with an outwardly stretched ligand-binding site. This mechanism differs from most transmembrane signaling molecules, which usually achieve activation through association with their target molecules.

To investigate how the transmembrane segment mediates these changes, Luo, Springer, and Takagi systematically replaced amino acids in both the α and β transmembrane domains of the heterodimer with cysteines, creating the potential for binding interactions through a chemical reaction, disulfide bond formation, between the two subunits. By analyzing 120 possible cysteine pairs, the researchers not only confirmed the structure of the transmembrane region as helical but also mapped the proximal amino acid residues between the helices. To understand how the helical transmembrane domains transmit signals, the team introduced activating mutations in the amino acids of the α subunit cytoplasmic tail. Using this approach, they observed the loss of the contact between the subunits, indicating a separation of the transmembrane helices. Furthermore, when disulfide bond formation occurred, linking the transmembrane segments together, activation was suppressed. While previous models had proposed various modes of subunit movements, including hinge- and piston-like models, these results strongly support the notion that lateral separation of the subunits is the driving force behind the signal. As many diseases arise from defects in integrin adhesion, understanding the conformation and mechanism of integrin activation could suggest promising avenues for drug development aimed at correcting such defects.

